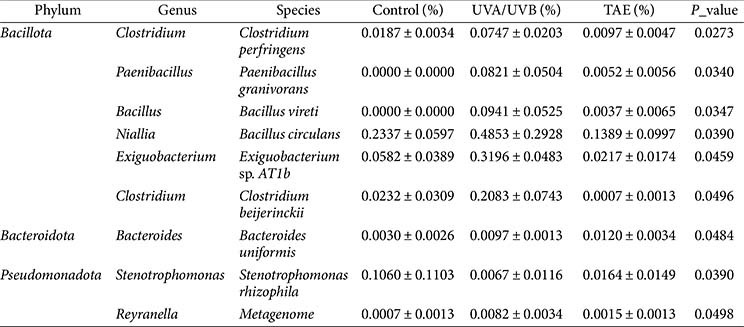# Protective and Reparative Effects of *Tremella aurantialba* Extract against Skin Photoaging and Its Underlying Mechanisms

**DOI:** 10.4014/jmb.2507.07053

**Published:** 2025-10-29

**Authors:** Guanjie Peng, Bowen Sun, Shangjun Gao, Jingjing Cheng, Sihui Chen, Guofeng Shi, Lanyue Zhang

**Affiliations:** 1 Fankol Biotechnology (Guangzhou) Co., Ltd., Guangzhou 510006, P.R. China; 2 School of Biomedical and Pharmaceutical Sciences, Guangdong University of Technology, Guangdong Provincial Key Laboratory of Plant Resources Biorefinery, Guangzhou 510006, P.R. China

**Keywords:** *Tremella aurantialba*, UVA/UVB, photoaging, metabolomics, microbiomics, transcriptomics

## Abstract

While *Tremella aurantialba* (*T. aurantialba*) is known for its significant antioxidant and anti-inflammatory activities, its role in skin photoaging remains unclear. In this study, we elucidated the protective mechanisms of *T. aurantialba* extract (TAE) against ultraviolet A and ultraviolet B (UVA/UVB)-induced skin photoaging. Using UHPLC-Q-Orbitrap-MS analysis, a total of 24 compounds were identified from TAE, including trigonelline (22.83%), isonicotinic acid (18.16%), acetylcholine (16.66%), choline (15.02%), and 2-hydroxyphenylalanine (6.85%). *In vitro* investigations revealed that TAE significantly enhanced the survival rate of UVB-induced HaCaT cells, promoted cell migration, and increased the migration rates to above 50%, while concurrently reducing reactive oxygen species (ROS) levels. *In vivo*, TAE suppressed abnormal epidermal thickening and mast cell infiltration induced by UVA/UVB in the dorsal skin of mice, and facilitated the restoration of collagen fibers. Metabolomics analysis indicated that TAE mitigated photoaging mainly by modulating the sphingolipid and glycerophospholipid metabolic pathways. Skin microbiome analysis showed that TAE boosts *Bacillota* and *Bacteroidota* while suppressing *Exiguobacterium* and *Lachnospiraceae*, consequently restoring skin microbiota balance and facilitating skin repair. Transcriptome analysis demonstrated that genes modulated by TAE were primarily concentrated in lipid metabolism, circadian rhythm, and response to oxygen-containing compounds. This suggests TAE facilitated skin barrier repair and mitigated UVA/UVB-induced photoaging by modulating cellular physiological rhythms and lipid metabolism, while controlling oxidative stress. In conclusion, TAE mitigates UVA/UVB-induced damage via multi-targeted antioxidant, anti-inflammatory, and skin barrier repair-promoting effects, thereby establishing a scientific basis for its application in developing functional foods and cosmetics to target photoaging.

## Introduction

Under prolonged exposure to ultraviolet (UV) radiation, the skin is susceptible to damage, which may induce symptoms associated with photoaging. UVA and UVB are the primary factors contributing to photoaging, as UVA can penetrate deeply into the upper layers of the dermis, while UVB can traverse the epidermis. Their combined effects disrupt the deeper dermal-epidermal junction, leading to increased accumulation of DNA damage, activation of inflammatory responses, and oxidative stress [1]. These chain reactions activate multiple signaling pathways, resulting in a reduction in collagen production [2]. Upon exposure to UV radiation, cells generate reactive oxygen species (ROS) that trigger inflammatory responses and cause damage to proteins within the dermis. At the macroscopic level, these changes manifest as an increase in epidermal thickness and wrinkles, resulting in dryness and hyperpigmentation, which are the manifestations of photoaged skin.

Studies have shown that monomeric polysaccharides in many traditional Chinese medicine ingredients have significant effects on the antioxidant activity of skin cells [3]. In addition, according to the “Compendium of Materia Medica,” *T. aurantialba* was used for protecting the liver, relieving cough, and moistening the lungs. This fungal species is rich in physiologically active substances, including *T. aurantialba* polysaccharides (TAP). Mushroom polysaccharides are natural moisturizing agents that are instrumental in improving the hydrating properties of cosmetic formulations [4]. The degradation products of TAP can also significantly promote the synthesis of aquaporin in epidermal keratinocytes, and show good water absorption and retention properties with remarkable moisturizing effects [5]. In addition, *T. aurantialba* fermented polysaccharides also possess strong antioxidant activity [6], while the water extracts can regulate intestinal flora [7]. The polysaccharides of *T. fuciformis*, another member of the same species, have also been shown to improve skin aging, but no studies have been done on its extracts [8]. Current research on *T. aurantialba* has primarily focused on TAP, while studies on its water extract are scarce. More crucially, there are no existing reports regarding the underlying mechanisms of *T. aurantialba*, or even whether it possesses anti-photoaging effects. Polysaccharides need to be obtained through more complicated steps, and if their simple extracts can produce the same or better results, it may benefit their application. Given that *T. aurantialba* has demonstrated significant anti-inflammatory and antioxidant activities, we aimed in this study to fill this gap by comprehensively exploring the impact of *T. aurantialba* extract (TAE) on photoaging and its mechanisms of action.

For this purpose, we utilized UHPLC-Q-Orbitrap-MS to analyze the components of TAE and employed UVB-induced HaCaT cells to construct an *in vitro* anti-photoaging model. The present research validated the anti-photoaging effect of TAE by assessing its impact on HaCaT cell migration and ROS levels at varying concentrations. After confirming the anti-photoaging potential of TAE *in vitro*, a mouse photoaging model induced by combined UVA and UVB was established. The repair effect of TAE on skin tissue was evaluated through hematoxylin and eosin (HE) staining, toluidine blue (TB) staining, and Masson staining. Transcriptome and metabolome analyses in addition to skin microbiome community analysis were conducted to assess the gene expression, metabolic pathways, and skin flora changes induced by TAE, and to explore its anti-photoaging mechanisms. Finally, we sought to reveal in this study how TAE combats UV-induced damage with the goal of providing scientific evidence for its development and utilization in anti-photoaging functional cosmetics.

## Materials and Methods

### Reagents

Human immortalized keratinocyte cells (HaCaT) were purchased from Wuhan Bodex Bioengineering Co., Ltd.(China) and stored in laboratory liquid nitrogen tanks. The DCFH-DA probe and Methyl-thiazolyl-tetrazolium (MTT) were purchased from Beijing Solebo Technology Co., Ltd. (China). The remaining chemical reagents were of analytical grade and were supplied by Aladdin Reagent Co., Ltd. (China). PBS buffer, FBS fetal bovine serum, high-glucose DMEM medium, trypsin, and penicillin-streptomycin were purchased from Gibco (USA).

### Extraction of *T. aurantialba*

*T. aurantialba* (5 kg) was collected from Yunnan Province. The plant was identified by Professor Nian Liu (Zhongkai University of Agriculture and Engineering, China). Some specimens, serving as vouchers, are currently preserved at the Institute of Natural Medicine and Green Chemistry (Guangdong University of Technology, China). The voucher specimen number is 2024-111T. *T. aurantialba* was pulverized and sieved through a 40-mesh sieve. Subsequently, 10 g of *T. aurantialba* was weighed out and placed into a round-bottom flask, to which 300 ml of water was subsequently added. The mixture was then heated and refluxed in a 90°C water bath for 4 h [7]. After filtration, the filtrate was concentrated to a paste and vacuum freeze-dried for 48 h to obtain the TAE, which was then stored at -20°C for subsequent use.

### Analysis of TAE Components

**Fig. 1:**
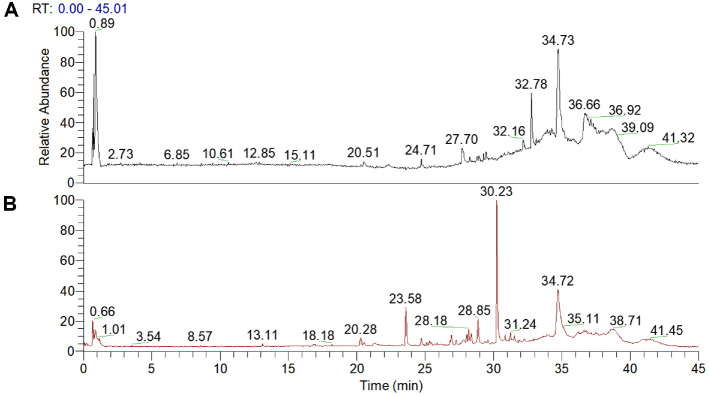
Total ion chromatograms of *Tremella aurantialba* extract in positive (A) and negative (B) modes.

The components of TAE were examined using UHPLC-Q-Orbitrap-MS (Thermo, Ultimate 3000LC, Q Exactive HF, USA) with a C_18_ column (Zorbax Eclipse, USA). Separation involved 40°C column temp, 10°C sample manager, 5 μl injection, 0.3 ml/min flow, and a gradient elution (A: 0.1% formic acid, B: acetonitrile). Mass spectra were acquired in positive and negative modes (m/z 50-1000), with specific ion source parameters. Data were preliminarily processed using Thermo Xcalibur 4.1, and compounds were identified by matching exact masses with theoretical values using the mz-Cloud database [9]. The total ion chromatograms of TAE in positive a nd negative modes are shown in Fig. 1.

### Cell Viability Assay

HaCaT cells were cultured with DMEM containing 10% FBS in a cell incubator at 37°C, 5% CO_2_. The logarithmic phase HaCaT cells were digested with trypsin and spun down. Cells were resuspended in complete medium and adjusted to a concentration of 8 × 10^4^ cells/ml. Subsequently, 100 μl of the cell suspension was added to each well of a 96-well plate. After incubation in a cell culture incubator for 24 h, the medium was discarded, and 100 μl of diluted TAE at different concentrations (12.5-800 μg/ml) was introduced into a 96-well plate. All experimental groups, with the exception of the control group, were subjected to UVB radiation (wavelength 280-320 nm) at an irradiation dose of 40 mJ/cm^2^. After 24 h of incubation, the original liquid was discarded, and 100 μl of 0.5 mg/ml MTT solution was added to each well. After further incubation for 4 h, the MTT solution was carefully discarded, 100 μl of DMSO was introduced into each well, and then subjected to agitation on a shaker for 10 min. Then, the 96-well plate was placed on a microplate reader and the absorbance (OD) was measured at a wavelength of 570nm [9]. The number of wells in each experimental group was six. The cell viability is calculated according to the formula:


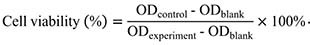


### HaCaT Cell Migration Assay

Cells were seeded at a density of 5 × 10^5^ cells per well in 24-well plates with 1 mL of medium. After the cells had adhered and reached confluence, a scratch wound was created using a 200 μl pipette tip held vertically. The wells were then thoroughly washed with PBS to remove the detached cells, which were subsequently discarded. Except for the control group, all other groups were irradiated using a UVB lamp (wavelength: 280-320 nm) at a dose of 40 mJ/cm^2^ to establish a photoaging model. Following the modeling procedure, the PBS in the wells was aspirated. The control and model groups were replenished with 1 ml of fresh culture medium, while the treatment groups received 1 ml of DMEM containing TAE at concentrations of 50, 100, or 200 μg/ml. All groups were cultured in medium without serum and antibiotics, aiming to minimize the influence of cell proliferation on the experimental outcomes. Images of the scratch wounds were captured under an inverted microscope at 0 and 24 h [9], and the migration rate was calculated using a specific formula:


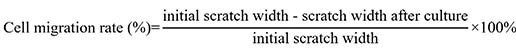


### ROS Detection

Cells were seeded at a density of 5 × 10^5^ cells per well in 24-well plates. After 24 h, all groups except the control group were irradiated using a UVB radiometer at a dose of 40 mJ/cm^2^. Following irradiation, PBS was aspirated from the wells. The control and model groups were replenished with 1 mL of fresh culture medium, while the treatment groups received 1 ml of DMEM containing TAE at concentrations of 50, 100, and 200 μg/ml, respectively. After 24 h, the cells were washed three times with PBS. Then, a DCFH-DA probe at a concentration of 10 μM diluted with PBS was added and reacted for 30 min. After removal of the probe, cells were washed with PBS three times for 5 min each, and the images were observed using an inverted fluorescence microscope in the dark [10]. The OD values in the images were quantified using Image Pro Plus 6.0 software.

### Animal Experiments

SPF-grade KM mice (male, weighing 34-38 g, 5 weeks old) were purchased from the Guangzhou Rui Ge Biotechnology Co., Ltd. China (SCXK(Yue)2023-0059). The mice were randomly divided into a control group (Control), a model group with UVA and UVB irradiation (UVA/UVB), a 0.25% TAE group (TAE-L), a 0.5% TAE group (TAE-M), and a 1% TAE group (TAE-H) [11, 12], with 6 mice in each group. An approximately 4 × 4 cm area on the backs of the mice was cleared of hair. Except for the blank group, UVA combined with UVB was used to simulate sunlight irradiation. The irradiation dose ratio of UVA to UVB was 10:1, with the total irradiation dose set at UVB = 0.1 J/cm^2^ and UVA = 1 J/cm^2^ [13]. The light source was approximately 15 cm above the skin surface, and irradiation was performed once daily, with an interval of one day between sessions, lasting for 4 weeks. Immediately after irradiation, drug treatment was administered by applying 200 μl of the drug topically to the dorsal skin of each mouse. Water and food were then provided, and the mice were allowed free movement. Twenty-eight days after the final drug administration, the mice were euthanized, and the dorsal skin at the drug application sites was excised for further analysis.

### Hematoxylin and Eosin Staining

The skin tissue of the target animal was embedded in paraffin, fixed, and sliced. The paraffin sections were then de-waxed and rehydrated. Subsequently, HE staining was performed according to the manufacturer’s instructions. Following dehydration and mounting, the sections were examined microscopically, and images were acquired and analyzed [14].

### Toluidine Blue Staining

The paraffin sections were de-waxed in water, and the animal tissue sections were placed in dye solution (2~5 min). After rinsing them with water to remove residual dye, the samples were differentiated with 0.1% glacial acetic acid, and sealed transparently after drying. The final samples were then sent for microscopic examination, and images were collected and analyzed [14].

### Masson Staining

Paraffin sections were de-waxed with water, fixed with Bouin's solution, stained with sky blue for 2-3 min, washed, stained with hematoxylin staining solution for 2-3 min, differentiated with 1% hydrochloric acid ethanol for a few seconds, soaked in rinse for 10 min, and then stained with fuchsin solution. This was followed by microscopic examination, image acquisition, and analysis [14].

### Metabolomics Analysis

Mouse dorsal skin (0.1 g) was homogenized in 1 ml of methanol at 4°C. After incubation for 10 min and centrifugation (4°C, 13,000 ×*g*, 15 min), the supernatant was filtered (0.22 μm) and analyzed by UHPLC-MS. Separation was performed on an Agilent 1290 Ultra High-Performance Liquid Chromatography (UPLC) system with a hydrophilic interaction liquid chromatography (HILIC) column (25°C, 0.5 ml/min, 2 μl injection), employing a gradient of water/ammonium acetate/ammonia (A) and acetonitrile (B). An AB Sciex TripleTOF 6600 Mass Spectrometer was used to acquire MS and MS/MS spectra (ESI ± 5500 V, m/z 60-1000, product ions 25-1000 Da). The clustering potentials of both positive and negative modes were ± 60 V, and the collision energy was (35 ± 15) eV [15].

### Transcriptome Analysis

For transcriptome sequencing of library data and analysis, the Illumina HiSeq 4000 platform was used. Low-quality data was filtered out of the original data by a fast quality control method to ensure data quality. This includes the removal of sequences containing linkers, sequences containing more than 10% uncertain bases (N), sequences containing base A, and sequences containing more than 50% low-quality bases (Q ≤ 20). Ribosomal alignment, sequencing sequence alignment and reference genome alignment, transcript reconstruction (calculating the expression of all genes in each sample), sample correlation analysis, and differentially expressed gene analysis (labeling FDR < 0 and | log2(FC) | > 1 as genes with significant differences) were analyzed by short sequence alignment tools Bowtie2, HISAT2, Stringtie, R language, and DESeq [16]. The differentially expressed proteins were compared and tested one by one using various terms in the Gene Ontology (GO) database, and the differentially expressed genes were condensed and analyzed by GO. In addition, the sequencing results were compared with the Kyoto Encyclopedia of Genes and Genomes (KEGG) database, and the differentially expressed genes were functionally annotated and classified.

### Skin Microbiome Analysis

Total microbial DNA was extracted from skin swab samples according to the instructions provided with the E.Z.N.A. Tissue DNA Kit (Omega Bio-tek, Norcross, USA). The quality of the extracted genomic DNA was assessed by 1% agarose gel electrophoresis, and the DNA concentration and purity were measured using a NanoDrop 2000 (Thermo Scientific, USA). Using the extracted DNA as a template, the V3-V4 variable region of the 16S rRNA gene was PCR-amplified with the forward primer 338F (5’-ACTCCTACGGGAGGCAGCAG-3’) and the reverse primer 806R (5’-GGACTACHVGGGTWTCTAAT-3’), both of which contained a barcode sequence. The PCR products were recovered from a 2% agarose gel and then purified using a DNA Gel Extraction Purification Kit (PCR Clean-Up Kit, China). The concentration of the purified products was quantified using a Qubit 4.0 fluorometer (Thermo Scientific). Library preparation was performed on the purified PCR products using the NEXTFLEX Rapid DNA-Seq Kit. Sequencing was carried out on the Illumina NextSeq 2000 platform by Shanghai Majorbio Bio-pharm Technology Co., Ltd. China. The raw paired-end sequencing reads were quality-controlled using fastp software (https://github.com/OpenGene/fastp, version 0.19.6) and then merged using FLASH software (http://www.cbcb.umd.edu/software/flash, version 1.2.11). The quality-filtered and merged sequences were clustered into operational taxonomic units (OTUs) at a 97% similarity level using UPARSE v7.1 software (http://drive5.com/uparse/), and chimeric sequences were removed during this process. To minimize the impact of sequencing depth on subsequent alpha and beta diversity analyses, the number of sequences in all samples was rarefied to 20,000. After rarefaction, the average Good’s coverage for each sample remained at 99.09%. For taxonomic annotation, the RDP classifier (http://rdp.cme.msu.edu/, version 2.11) was used to compare the OTUs against the Silva 16S rRNA gene database (v138) with a confidence threshold of 70%. The community composition of each sample was then summarized at various taxonomic levels. Functional prediction analysis of the 16S data was performed using PICRUSt2 software (version 2.2.0).

All data analyses were conducted on the Majorbio Cloud Platform (https://cloud.majorbio.com). The mothur software (http://www.mothur.org/wiki/Calculators) was used to calculate alpha diversity indices, including Chao1 and Shannon. The Wilcoxon rank-sum test was employed to analyze inter-group differences in alpha diversity. Principal Coordinates Analysis (PCoA) based on the Bray-Curtis distance algorithm was used to assess the similarity of microbial community structures among samples. Linear discriminant analysis Effect Size (LEfSe) analysis (http://huttenhower.sph.harvard.edu/LEfSe) (LDA>2, *p* < 0.05) was performed to identify bacterial taxa with significantly different abundances between groups, from the phylum to the genus level. Distance-based redundancy analysis (db-RDA) was used to investigate the factors influencing the skin bacterial community structure. Linear regression analysis was used to assess the impact of key clinical variables, identified in the db-RDA, on the microbial alpha diversity indices. For correlation network analysis, species were selected based on a Spearman correlation coefficient |r| > 0.6 and a *p*-value < 0.05 [17, 18].

### Data Processing

Data were presented as the mean ± standard error. All statistical analyses were performed using GraphPad Prism 8.0.2 statistical software. One-factor analysis of variance was used to compare the means of multiple groups. A two-sided Student *t*-test was used for pairwise comparison between small groups. A *p*-value < 0.05 was considered statistically significant (*p < 0.05), indicating a significant difference compared with the UVA/UVB group.

## Results

### Analysis Results for TAE Components

A total of 24 compounds were identified in TAE (Table 1), including trigonelline (22.83%), isonicotinic acid (18.16%), acetylcholine (16.66%), choline (15.02%), and 2-hydroxyphenylalanine (6.85%). Trigonelline, as an antioxidant and anti-inflammatory agent, can counteract inflammation at multiple levels by inhibiting pro-inflammatory factor release, mitigating the spread of inflammation, and reducing tissue damage [19]. Isonicotinic acid and its derivatives are potent anti-inflammatory compounds and also ROS inhibitors [20]. Both acetylcholine and choline possess anti-neuroinflammatory effects, demonstrating certain anti-inflammatory potential [21]. Additionally, TAE contains L-ergothioneine and D-(-)-mannitol. L-Ergothioneine is an effective cytoprotective and antioxidant agent [22], while D-(-)-mannitol has been reported to alleviate inflammatory edema, airway inflammation, and intestinal inflammation [23], showing certain anti-inflammatory potential. Therefore, the anti-inflammatory potential of TAE may arise from the combined action of these components enabling it to exert its anti-photoaging effects.

### Effect of TAE on HaCaT Cell Viability

**Fig. 2:**
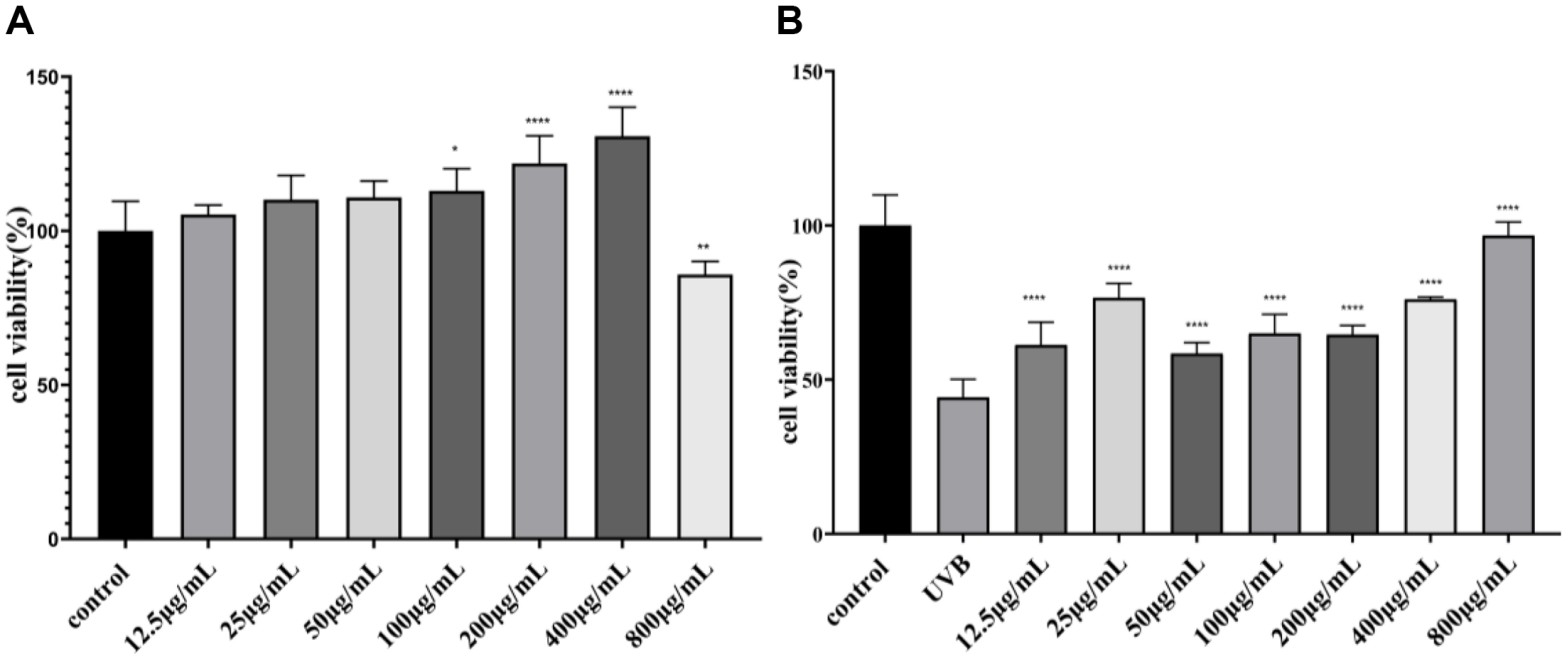
Effect of *Tremella aurantialba* extract on HaCaT cell viability. (**A**) Cytotoxicity assay of TAE on HaCaT cells under non-irradiated conditions, showing no significant toxicity within 12.5–400 µg/ml. (**B**) Cell viability of UVB-irradiated HaCaT cells after treatment with various concentrations of TAE (12. 5–800 µg/ml). TAE significantly improved cell survival in a dose-dependent manner compared with the UVB group. (vs UVB group, *p <0.05, **p <0.01, ****p <0.0001) Data showed mean ± SD.

As shown in Fig. 2A, TAE of 12.5-400 μg/ml showed no toxicity to the cells and could be used in subsequent experiments. Furthermore, as shown in Fig. 2B, UVB treatment could significantly reduce the cell survival rate (*p* < 0.0001), and the cell survival rate of the 12.5 μg/ml-800 μg/ml TAE group was markedly higher than that observed in the UVB group (*p* < 0.0001), which indicated that within the concentration range of 12.5 μg/ml-100 μg/ml, the cell survival rate of the TAE group was significantly higher than that of the UVB group (*p* < 0.0001). TAE was not toxic to HaCaT cells, and the high concentration of TAE could promote cell proliferation, indicating that TAE had the potential to protect against UVB damage. Subsequent experiments were carried out at concentrations of 50 μg/ml to 200 μg/ml.

### Reparative Capacity of TAE for Photoaged Cells

**Fig. 3:**
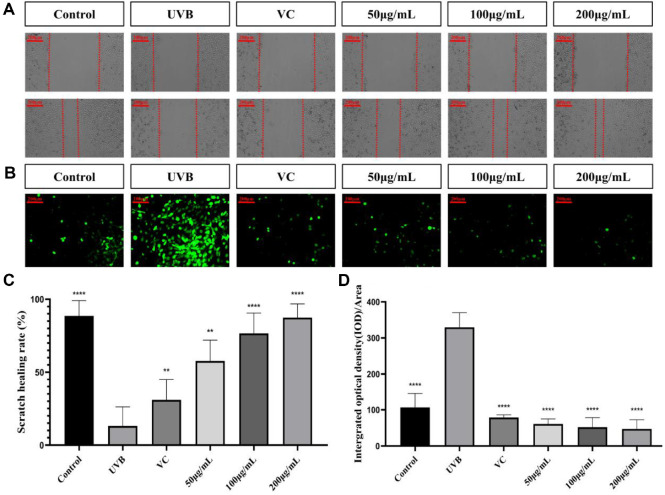
Effect of *Tremella aurantialba* extract on HaCaT cell migration for 24 h and ROS levels in HaCaT cells. (**A**) Scratch migration images of HaCaT cells extracted from the *Tremella aurantialba* extract in each group. (**B**) Immunofluorescence images of ROS production in each group in the presence of DCFH-DA (Green fluorescence reflects ROS levels). (**C**) Statistical results of the scratch healing rate of *Tremella aurantialba* extract in each group. (**D**) Quantitative analysis of ROS images. (vs UVB group, **p < 0.01, ****p < 0.0001) Data showed mean ± SD.

Due to the significant similarities between the mechanisms of skin photoaging and wound healing, it can be speculated that skin aging resembles a chronic and widespread "wound" that overwhelms the skin's inherent repair mechanisms. The migration of keratinocytes is crucial for re-epithelialization after both superficial and deeper skin injuries. Therefore, using a cell migration assay to assess the promotional impact of drugs on wound healing can indirectly reflect their potential therapeutic effects on photoaging. Fig. 3A and 3C showed that in comparison to the control group, the migration ability of cells in the UVB group was markedly reduced (*p* < 0.0001), with a migration rate of only 13.07%. After treatment with TAE, the cell migration rates at concentrations of 50 μg/ml, 100 μg/ml, and 200 μg/ml were 57.78%, 76.59%, and 87.43%, respectively. These rates were markedly greater than those observed in the UVB group and increased with escalating TAE concentrations. All three concentrations were more effective than the VC group (30.92%). The best effect of cell proliferation was observed in the 200 μg/ml group. The findings from the migration scratch assay indicated that TAE had a potential skin wound-healing effect, and the effect was better at high concentrations.

### Effect of TAE on ROS Release from HaCaT Cells

As illustrated in Fig. 3B and 3D, the UVB group showed a markedly increased ROS level compared to the control group (*p* < 0.0001). The level of ROS was significantly decreased (*p* < 0.0001) following treatment with TAE. In conclusion, TAE effectively reduces ROS levels within HaCaT cells, and the inhibitory effect is better with the increase of the concentration of TAE. Moreover, the effects of three concentrations of TAE are better than those of the VC group, and the effect of the 200 μg/ml treatment is the most significant.

### Effect of TAE on UVA/UVB-Induced Skin Hyperplasia

**Fig. 4:**
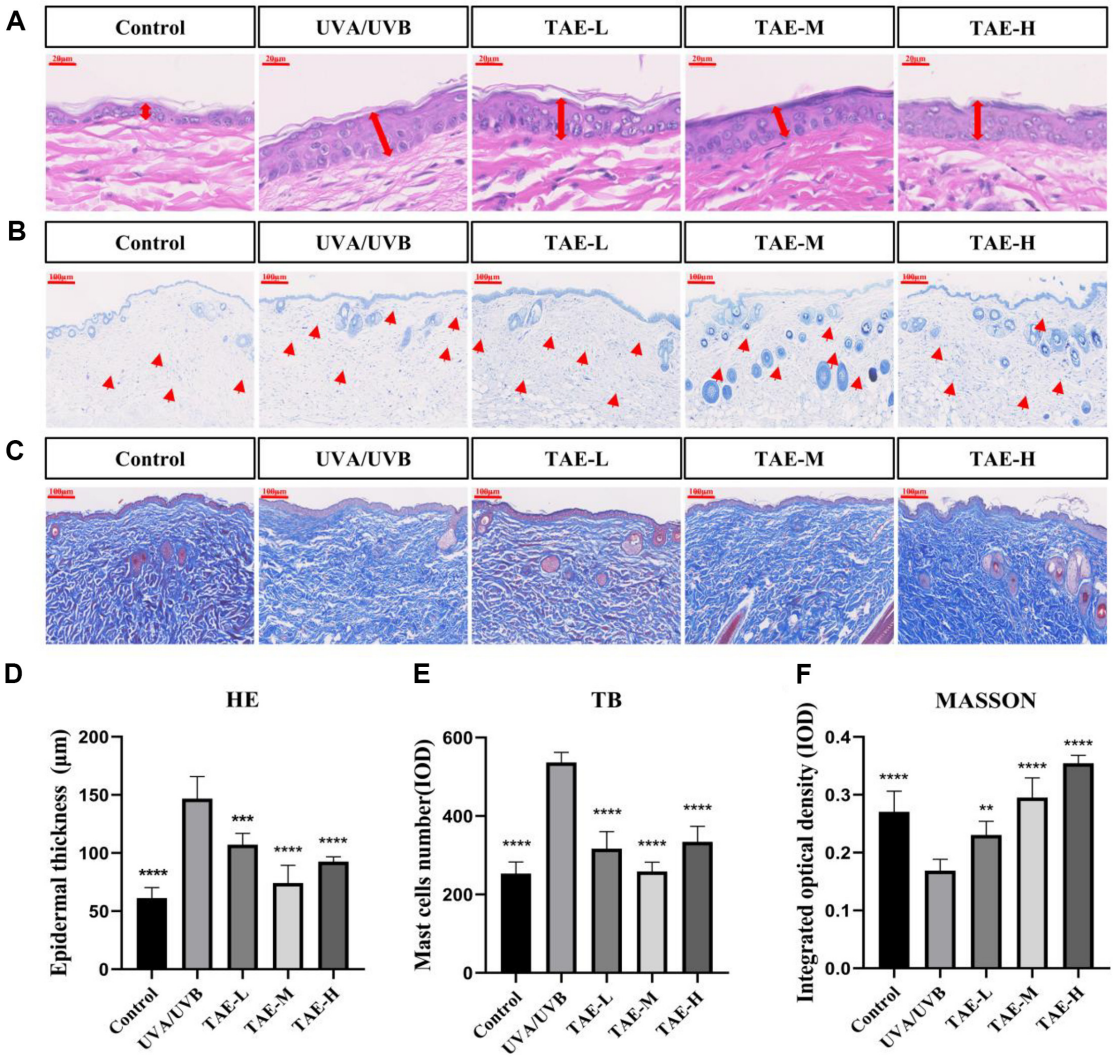
Effects of TAE on histomorphological changes, mast cell content, and collagen fiber in UVA/UVBdamaged mouse skin. (**A**) HE staining showing epidermal and dermal morphology. (**B**) TB staining visualizing mast cells. (**C**) Masson staining showing collagen fibers. (**D**) Quantification of epidermal thickness. (**E**) Quantification of mast cell numbers. (**F**) Collagen volume fraction analysis. (Model group: UVA/UVB, Treatment groups: TAE-L, TAE and TAE-H containing 0.25%, 0.5%, and 1% TAE respectively, *** *P* < 0.01 and ****P < 0.0001 indicate statistically significant differences from the model group) Data showed mean ± SD (*n* = 6).

UV-induced skin damage can lead to increased roughness of the skin surface and abnormal thickening of the epidermis. These phenomena were further confirmed in the skin analysis characteristics of this study, as shown in Fig. 4A. Compared to the control group, the UVB group showed a markedly increased skin thickness after UV irradiation (*p* < 0.01) (Fig. 4D). After treatment, the skin problems of the experimental animals showed signs of significant improvement. The TAE-L, TAE-M, and TAE-H groups could slow down abnormal epidermal proliferation to some extent, with the TAE-M group showing the most significant effect.

### Effect of TAE on UVA/UVB-Induced Increase in Mast Cells

Abnormal mast cell proliferation is a key characteristic of skin inflammation. Additionally, skin inflammation accelerates the process of skin photoaging. Compared to the control and UVA/UVB groups, dermal mast cell counts were significantly and abnormally elevated in the UVA/UVB group. The infiltration of mast cells at different concentrations in the TAE treatment groups showed a decreasing trend, which was markedly lower than that in the UVA/UVB group, suggesting that TAE can help decelerate the excessive proliferation of mast cells resulting from inflammatory processes, and the decrease in mast cell count was most significant in the TAE-M group (Fig. 4B, 4D, and 4E).

### Effect of TAE on Skin Collagen Fibers

UV radiation can directly destroy skin collagen fibers. Therefore, the content of collagen fibers in the skin is often used as an indicator to evaluate the effectiveness of resistance to photo damage caused by UVA/UVB radiation. In the control group, the collagen fibers on the back showed a tight and orderly arrangement. However, in the UVA/UVB group, the collagen fibers were significantly reduced and had a disorganized arrangement, with some of them being broken (Fig. 4C). As shown in Fig. 4C and 4F compared to the UVA/UVB group, there was a significant rise in collagen fiber count within the TAE-treated groups (TAE-L, TAE-M, TAE-H). After TAE treatment, the integrated OD values significantly increased compared to the model group (Fig. 4F). Local application of TAE effectively repaired the collagen fiber damage caused by ultraviolet radiation.

### Screening of Differentially Expressed Metabolites (DEMs) in Skin Tissues

**Fig. 5:**
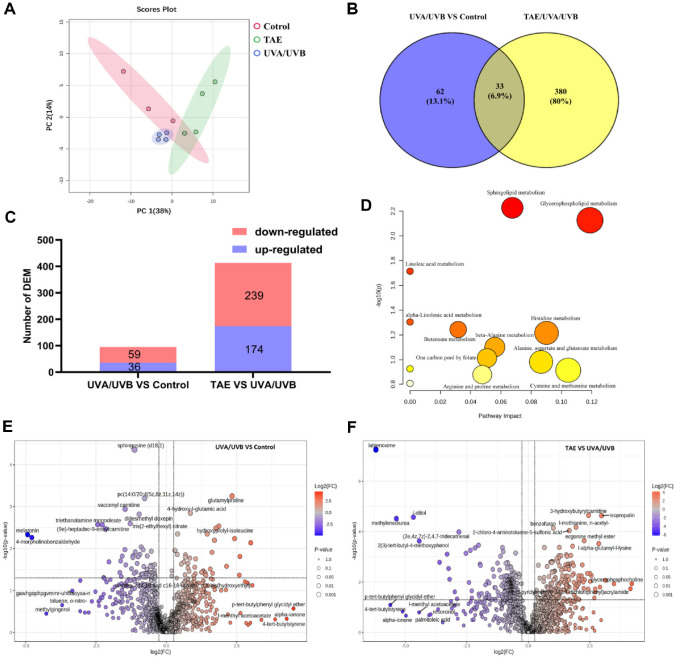
Meta bolomic profiling and pathway enrichment analysis of mouse skin tissue. (**A**) PCA graph. (**B**) The Venn plot of DEMs. (**C**) Number of DEMs in different groups. (**D**) Metabolic pathway map of mice (The darker the red color of the circle, the stronger its significance, and the larger the diameter, the more metabolites are enriched). (**E-F**) Volcano plot of metabolic differential in mice (Red circles represent upregulation, blue circles represent downregulation, and darker colors indicate more significant up/downregulation).

**Fig. 6:**
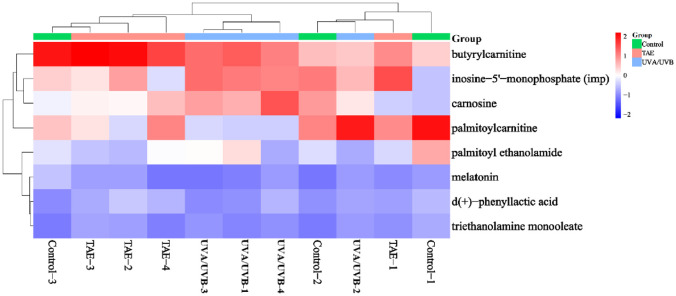
Heat map of clustering of common DEMs (Red represent upregulation, blue circles represent downregulation, and darker colors indicate more significant up/downregulation).

Unsupervised (principal component analysis) PCA is widely used for data exploration, visualization, compression, and feature extraction. All samples were analyzed using this method. Fig. 5A demonstrates that samples within each group clustered together, suggesting good reproducibility among the samples within those groups. To identify differentially expressed metabolites (DEMs) between different comparison groups, pairwise comparisons were conducted for each group. Compared to the control group, the UVA/UVB group showed 36 upregulated and 59 downregulated DEMs. Relative to the UVA/UVB group, the TAE group exhibited 174 upregulated and 239 downregulated DEMs (Fig. 5C), indicating that TAE can affect more metabolites in regulating skin inflammation. Venn analysis was conducted on these 413 DEMs with the 95 DEMs from the control and UVA/UVB groups, and 33 common DEMs (Fig. 5B). Through MetaboAnalysis, 13 endogenous metabolites were selected (Fig. 6). Compared with the control group, the palmitoylcarnitine and d(+)-phenyllactic acid were downregulated. The melatonin, butyrylcarnitine, inosine-5'-monophosphate (IMP), triethanolamine monooleate, palmitoyl ethanolamide, and carnosine were upregulated. However, treatment with TAE reversed the changes in most of these, indicating that TAE possesses some ability to modulate metabolic disorders.

### Metabolic Pathway Analysis of the Effect of TAE on Skin Photoaging in Mice

Subsequently, the 13 DEMs were uploaded to MetaboAnalysis, and the analysis results are shown in Fig. 5D. The top 5 metabolic pathways are sphingolipid metabolism, glycerophospholipid metabolism, linoleic acid metabolism, alpha-linolenic acid metabolism, and butanoate metabolism.

As shown in Fig. 5E and 5F, the top five metabolites significantly upregulated in the control vs. the UVA/UVB group are 4-tert-butylstyrene, alpha-ionone, p-tert-butylphenyl glycidyl ether, I-methyl acetoacetate, and glutamylproline. The top five metabolites significantly downregulated are melatonin, 4-morpholinobenzaldehyde, triethanolamine monooleate, (9e)-heptadec-9-enoylcarnitine, and methylgingerol. In the TAE vs. UVA/UVB group, the top five metabolites significantly upregulated are glycerophosphocholine, I–alpha–gutamyl–lysine, ecgonine methyl ester, 3-hydroxybutyrylcarnitine, and isopropalin; the top five metabolites significantly downregulated are p-tert-butylbenzyl glycidyl ether, methylenediurea, I-iditol, 4-tert-butylstyrene, and alpha-ionone.

### Analysis of Skin Microbiota in Photoaged Mice

**Fig. 7:**
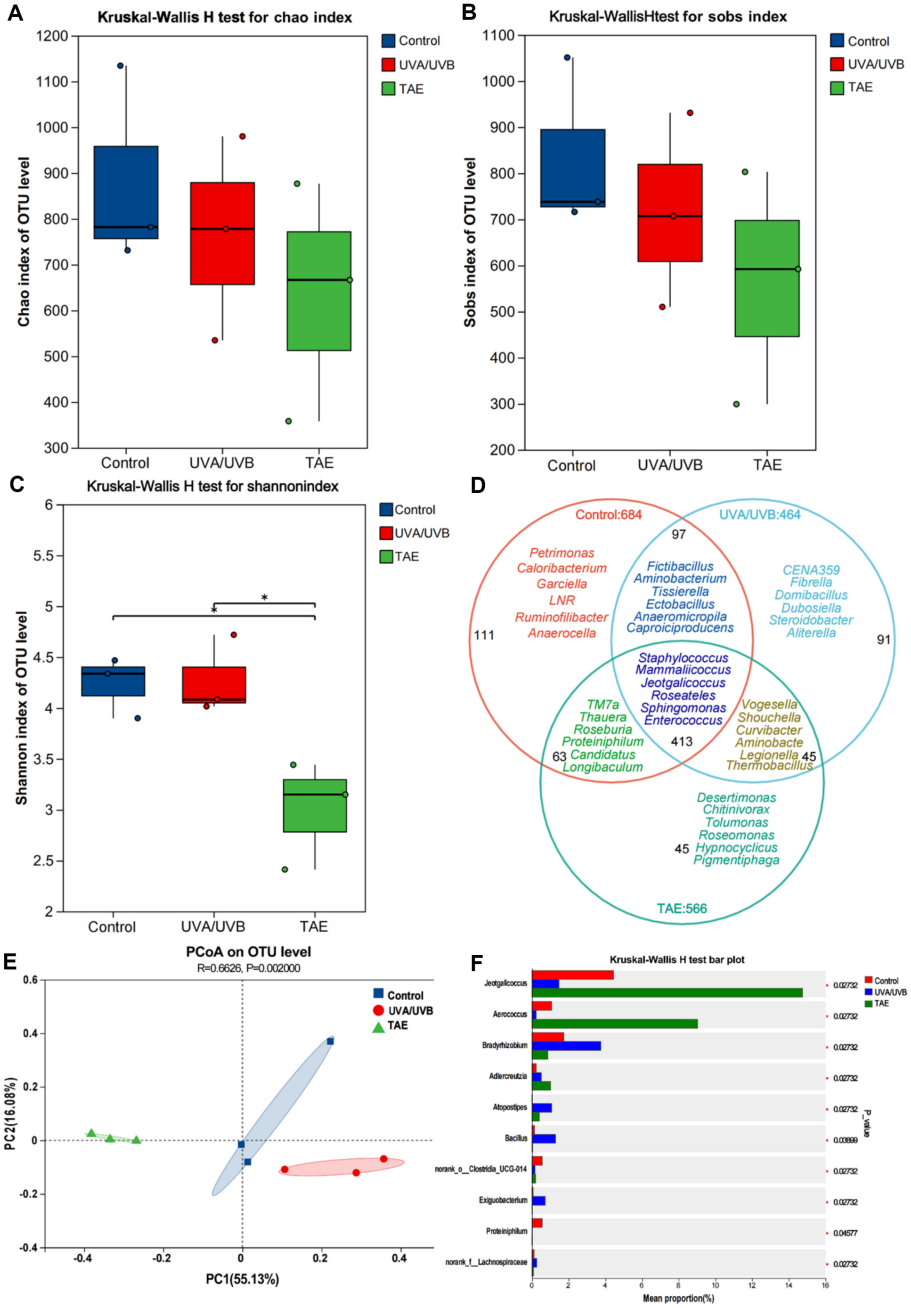
Effect of Tremella aurantialba extract on skin microbial diversity and composition. (**A**) Chao index. (**B**) Sobs index. (**C**) Shannon index. (**D**) Venn diagram at the genus level. (**E**) PCoA of bacterial samples at the OTU level between different groups. (**F**) Bar graph of the Wilcoxon rank-sum test.

The microbial community in the control group exhibits the highest alpha diversity, while the alpha diversity of the microbial community in the UVA/UVB group is reduced compared to the control group, and the reduction in the TAE group is even more significant (Fig. 7A-7C). The Venn diagram (Fig. 7D) shows that the proportion of shared genera among the three groups reaches 50.06%. The main bacterial groups include *Staphylococcus*, *Enterococcus*, *Mammallicoccus*, *Streptococcus*, *Roseateles*, and *Acinetobacter*. Principal coordinate analysis (PCoA)(Fig. 7E) clearly shows that the control and UVA/UVB groups are relatively close in the coordinate space, indicating that there is a small difference in their community structure. However, the TAE-treated skin deviates significantly, indicating that the community structure has undergone significant changes after TAE treatment. Fig. 7F presents the expression differences of the top 10 genera in different groups. The number of *Exiguobacterium* and *Lachnospiraceae* after UVA/UVB exposure is significantly increased compared to the control group, while the TAE group is significantly reduced, even lower than the control group.

**Fig. 8:**
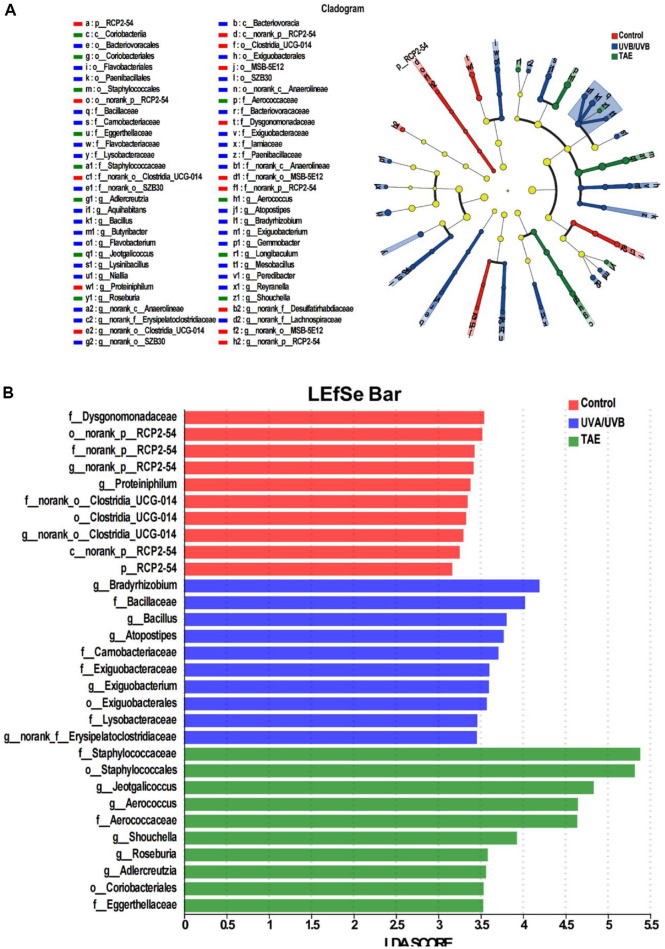
LEfS eanalysis of differential skin microbiota among groups. (**A**) Multi-level species hierarchy of LEfSe (yellow nodes represent no significant differences in microbial communities). (**B**) LEfSe (LDA effect size) analysis (A LDA> 3 is considered significant).

LEfSe analysis depiction (Fig. 8A–8B) reveals discrepancies in bacterial population densities across three groups at the taxonomic levels of phylum, genus, and species. The LDA effect size distribution (Fig. 8B) further highlights the key taxa contributing to these differences. The control group is enriched with Spirochaetota, Gallionella, and RCP2-54, among others. The UVA/UVB group is enriched with *Exiguobacterium*, *Exiguobacterales*, *Exiguobacteraceae*, *Niallia*, *Lachnospiraceae*, *SZB30*, *Pleomorphomonadaceae*, and *Ruminococcaceae*. These microorganisms are mainly associated with the environment. In contrast, the microbial composition of the TAE group is closely related to skin health, including *Verrucomicrobia*, *Roseburia*, and *Verrucomicrobiales*. This result suggests that topical application of TAE treatment can increase the number of beneficial skin bacteria and promote positive succession of the skin microbiome.

**Fig. 9:**
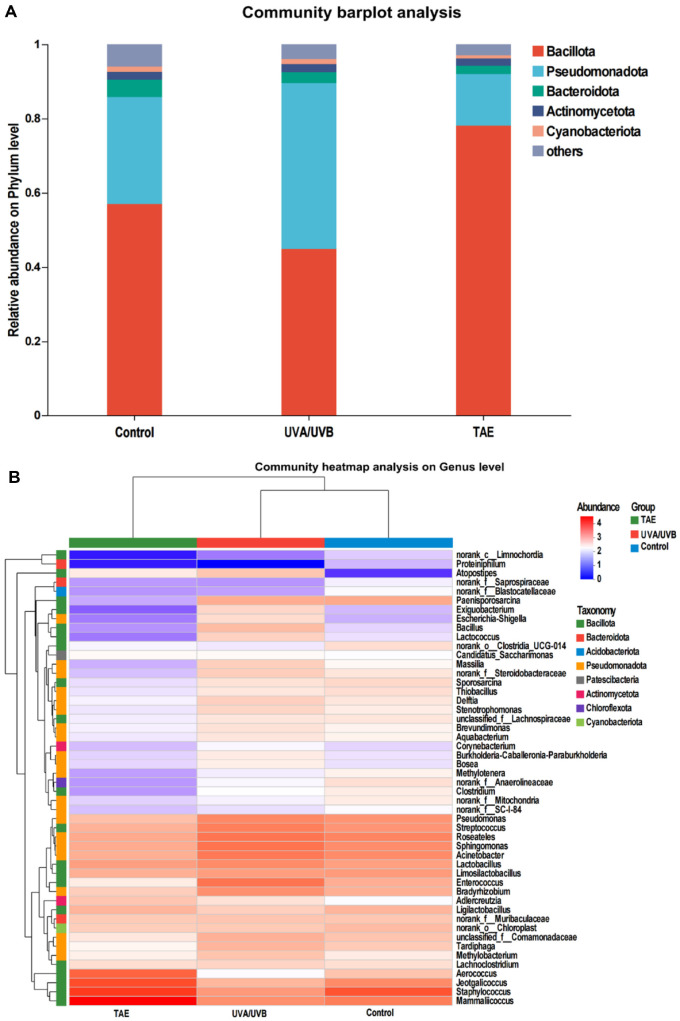
Effect s of TAE on skin microbiota composition at the phylum and genus levels. (**A**) Columnar accumulation diagram at the door level. (**B**) cluster heat map analysis at the genus level.

The combined analysis of Fig. 9A–9B and Table 2 demonstrates that TAE has a marked effect on the structure of the skin microbiome. Fig. 9A illustrates the compositional distribution at the phylum level, showing that in the control group, *Bacillota* dominates (56.99%), followed by Pseudomonadota (28.79%) and *Bacteroidota* (4.70%). After UVA/UVB exposure, *Bacillota* decreases to 44.92%, *Pseudomonadota* increases to 44.62%, and *Bacteroidota* decreases to 2.92%; whereas after TAE treatment, *Bacillota* increases to 53.43%, *Pseudomonadota* decreases to 23.41%, and *Bacteroidota* increases to 7.73%. The genus-level clustered heatmap in Fig. 9B also indicates that in the UVA/UVB group, genera such as *Staphylococcus*, *Jeotgalicoccus*, *Ligilactobacillus*, and *Mammalicoccus* showed a downward trend, while the relative abundance of inflammation-related genera such as *Enterococcus*, *Acinetobacter*, *Pseudomonas*, and *Streptococcus* increases, and the TAE group shows the opposite trend. Table 2 further provides the relative abundance and *p*-values of specific species, such as a significant reduction of metagenome in the *Pseudomonadota* phylum in the TAE group (*p* = 0.0498). These results collectively indicate that the TAE effectively regulates the skin microbiome and improves the skin microbiota.

### Transcriptome Analysis of UV-Damaged Mouse Skin Tissues by TAE

**Fig. 10:**
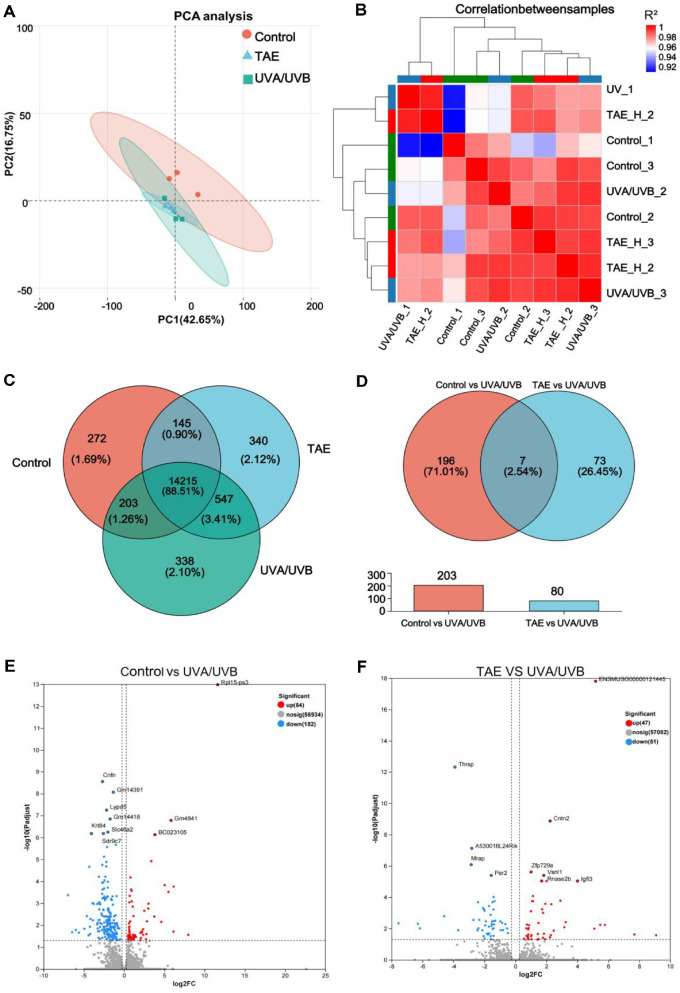
Trans criptomic analysis of UV-induced skin photoaging and TAE intervention. (**A**) PCA Results. (**B**) Sample correlation. (**C-D**) Venn diagram. (**E-F**) Volcanic map (Red represent upregulation, blue circles represent downregulation, and darker colors indicate more significant up/downregulation).

PCA was employed (Fig. 10A) to perform analysis on the degree of difference among various groups. The x-axis contributes 42.65%, and the y-axis 16.75%, primarily comparing the degree of sample aggregation through the x-axis. The repeatability and similarity among samples in each group, the control group, and the model group are all good. Fig. 10C shows an inter-group Venn analysis, with 145 common genes present in both the TAE and control groups, implying they belong to the photoaging model. Fig. 10C-10D presents a Venn analysis on the differentially expressed genes (DEGs), with a total of 80 genes in TAE vs. UVA/UVB, accounting for 26.45% of all groups, indicating a higher degree of gene differentiation in this set. As shown in Fig. 10E-10F, a total of 47 upregulated and 51 downregulated genes were detected in the TAE, and a total of 64 upregulated and 182 downregulated genes were detected in the control group. The significantly upregulated genes in the TAE group include *cntn2*, *zfp729a*, *Rnaselb*, *Igfl3*, *Vsnl1*, *Rnase2b*, and the downregulated genes include *thrsp*, *Mrap*, and *Per2*.

**Fig. 11:**
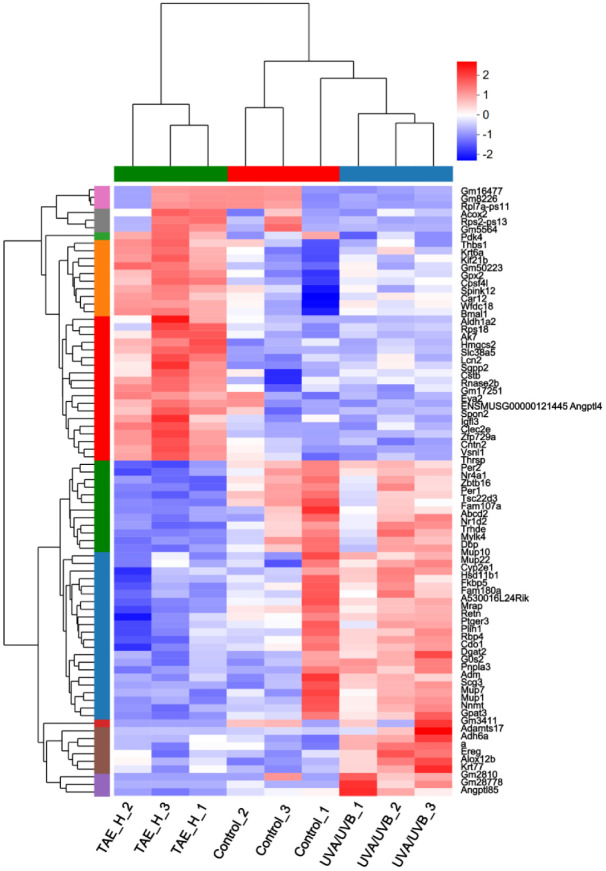
Heat map of taxa between TAE, UVA/UVB, and Control (Red represent upregulation, blue circles represent downregulation, and darker colors indicate more significant up/downregulation).

The expression pattern cluster analysis of the selected gene set was performed in the RNA-seq clustering heatmap. As shown in Fig. 11, genes with high expression levels in the UVA/UVB group have lower expression levels in the TAE group. The markedly different genes enriched in the clustering heatmap are *Gm53189*, *Fbn2*, *St6gal2*, *Krt77*, *Gprin2*, *Flg2*, *Rnf227*, *Lipk*, *Card14*, and *Adh6a*.

**Fig. 12:**
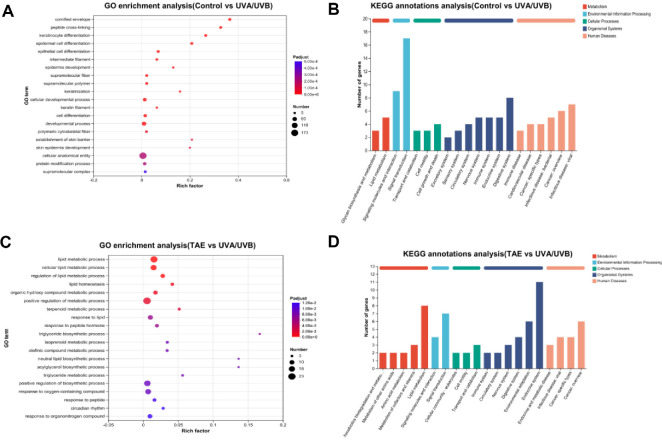
Funct ional enrichment analysis of DEGs between groups. (**A-B**) GO classification and KEGG enrichment analysis between the Control and UVA/UVB. (**C-D**) GO classification and KEGG enrichment analysis between TAE and UVA/ UVB.

In the TAE vs. UVA/UVB groups (Fig. 12A-12B), KEGG pathways that are significantly different (*p* < 0.05) include cellular lipid metabolism, lipid metabolism, positive regulation of metabolism, organic hydroxy compound metabolism, terpenoid metabolism, response to peptide hormone, triglyceride biosynthetic process, triglyceride metabolism, circadian rhythm, response to oxygen-containing compounds, and response to organonitrogen compounds. Fig. 12C and 12D show the number and classification of upregulated and downregulated genes annotated to a specific pathway. The trend of annotated pathways in the TAE and control groups is similar.

## Discussion

Prolonged exposure to UV contributes to skin photoaging, and affected skin can exhibit inflammatory manifestations, including erythema, edema, and pain [24]. The primary mechanism underlying how UVB induces photoaging in HaCaT cells lies in the excessive accumulation of intracellular ROS [25]. The TAP has various functions such as antioxidation, anti-diabetes, and blood lipid-lowering. The polysaccharides derived from *T. aurantialba* possess lubricating, film-forming, moisturizing, and radiation-protective activities. Inhibiting the inflammatory response and using antioxidants represent viable approaches to photoaging prevention.

Inhibiting inflammatory responses and antioxidation can serve as a method to prevent photoaging. In this study, multiple anti-inflammatory compounds were identified in TAE [26, 27], such as trigonelline, isonicotinic acid, acetylcholine, choline, L-ergothioneine, D-(-)-mannitol, and docosanoic acid, which suggested the antioxidant and anti-inflammatory potential of TAE, aligning with the outcomes of subsequent anti-photoaging experiments.

In *in vitro* experiments, a photoaging model of HaCaT cells was established via UVB exposure. In the cytotoxicity test, assays revealed that TAE enhanced cell proliferation within a non-cytotoxic range, suggesting that TAE may promote wound healing. The survival rate of cells subjected to TAE treatment was markedly higher than that of the UVB group, suggesting its protective role against UVB damage. There are significant similarities between the mechanisms associated with skin photoaging and those involved in wound healing [28]. In this study, the migration capacity of HaCaT cells irradiated with UVB diminished, while the migration rate of UVB-irradiated fibroblasts markedly improved in the presence of 50-200 μg/ml TAE, indicating its potential to repair photoaged skin. The key mechanism by which UVB induces photoaging in HaCaT cells lies in the excessive accumulation of intracellular ROS [25]. This study shows that TAE at 50-200 μg/ml effectively lowers UVB-induced ROS accumulation in HaCaT cells, demonstrating not only its protective effect against oxidative damage but also its capacity to enhance cellular antioxidant activity.

A prominent feature of skin aging is epidermal thickening [29]. The dermis is rich in collagen fibers, which form a major component of the extracellular matrix (ECM). A key feature of photoaged skin is the degradation of collagen fibers in the ECM, which results in a loss of connective tissue and abnormal accumulation of elastic fibers, ultimately causing the formation of wrinkles [30]. UVA/UVB radiation activates mast cells, leading to their degranulation and the release of inflammatory mediators (*e.g.*, histamine and prostaglandins), which worsens the inflammatory response and oxidative stress linked to photoaging [31]. In this study, we found that TAE effectively reduces UVA/UVB-induced abnormal epidermal thickening of the epidermis and the UVA/UVB-induced collagen fiber degradation, thereby slowing the skin aging process. Additionally, TAE inhibits the increase of mast cells caused by UVA/UVB radiation, thereby reducing the inflammatory response.

Sphingolipid and glycerophospholipid metabolism are crucial for cellular membranes. Upon cellular activation under oxidative stress, phospholipids undergo oxidation, and their oxidized products exhibit pro-inflammatory effects. Linoleic acid and alpha-linolenic acid contribute to maintaining the skin's lipid barrier and regulating inflammatory responses, as they possess significant antioxidant properties. Their metabolic dysregulation leads to impaired skin barrier function, reduced elasticity, wrinkle formation, exacerbated inflammation, and diminished antioxidant capacity [32]. Metabonomic analysis indicates that TAE primarily mitigates photoaging by modulating metabolic pathways, such as sphingolipid metabolism, glycerophospholipid metabolism, linoleic acid metabolism, and alpha-linolenic acid metabolism, thereby suppressing skin inflammation and enhancing barrier function.

*Exiguobacterium* is associated with skin infections, including ulcers, black eschars, and blisters [33]. *Lachnospiraceae* exhibits a statistically significant correlation with psoriasis and chronic spontaneous urticaria [34, 35]. The PCoA results of OTU levels of bacterial samples between different groups showed that treatment with TAE significantly reduced the abundance of *Exiguobacterium* and *Lachnospiraceae*, suggesting that TAE application can mitigate skin infections. LEFSe feature distribution results showed that the microbial composition in the TAE group was closely linked to skin health, featuring increased levels of *Verrucomicrobia*, *Roseburia*, and *Verrucomicrobiales*. *Roseburia* is implicated in modulating skin inflammation and maintaining microecological balance [36]. The combined analysis of the differences in the composition and expression of the microbial flora at the phylum and genus levels showed that in mice exposed to UVA/UVB radiation and treated with TAE, the abundances of *Bacillota* and *Bacteroidota* increased, while *Pseudomonadota* decreased. Certain genera within *Bacillota* are beneficial for skin health. Previous studies demonstrated that oral administration of *Bacillus subtilis* effectively alleviated skin lesion development in atopic dermatitis [37]. *Pseudomonas* may exacerbate inflammation, leading to elevated epidermal water loss and impaired skin barrier function [38]. Ishikawa et al. reported that *Bacteroidota* exerted anti-inflammatory effects [39], indicating its potential role in immune regulation and skin health maintenance. In the TAE group, the relative abundances of genera associated with inflammation, including *Enterococcus*, *Acinetobacter*, *Pseudomonas*, and *Streptococcus*, decreased; these genera are often linked to inflammation or compromised skin barrier integrity [40]. Conversely, genera, including *Staphylococcus*, *Jeotgalicoccus*, *Ligilactobacillus* and *Mammalicoccus* increased. These findings suggest that TAE intervention may alleviate skin aging by modulating the levels of these specific bacterial genera.

Significant differences in gene expression were observed between the TAE and UVA/UVB groups, with the TAE group demonstrating upregulation of *cntn2*, *Rnaselb*, and *Igfl3*, and downregulation of *thrsp*, *Mrap*, and *Per2*. Among them, the expression of *Igfl3* is mainly limited to the skin, which can promote cell proliferation and inhibit psoriasis and atopic dermatitis [41]. The significantly different genes enriched in the TAE and UVA/UVB are *Gm53189*, *Fbn2*, *Card14*, and *Adh6a*. These genes are mostly involved in connective tissue structure, glycan metabolism, skin barrier, and immune-inflammatory regulation. Among them, *Card14* is particularly enriched in skin cells and is crucial for maintaining the skin barrier and defending against external pathogens, which can potentially lead to disease development by promoting keratinocyte proliferation and inflammatory responses [42]. In the comparison of TAE vs. UVA/UVB, KEGG pathways that are significantly different include lipid metabolic process, cellular lipid metabolic process, circadian rhythm, response to oxygen-containing compounds, and response to organonitrogen compounds. Several studies consistently indicate that aging is characterized by abnormalities in lipid metabolism and cellular lipid metabolism. Lipid metabolism can regulate linoleic acid metabolism, which is consistent with metabolomics findings [43].

In summary, TAE at various concentrations was shown to effectively repair UV-induced photoaging in both *in vitro* and *in vivo* models. Integrated metabolomics and transcriptomics analyses revealed that TAE alleviates photoaging through the modulation of skin barrier and inflammation-related pathways. Genes regulated by TAE were predominantly enriched in pathways associated with lipid metabolism and circadian rhythm-related regions, suggesting it promotes skin barrier repair and combats UVA/UVB-induced photoaging by reshaping cellular physiological rhythms and lipid metabolic balance. Additionally, skin microbiome analysis showed that TAE enhances *Bacillota* and *Bacteroidota* while inhibiting *Exiguobacterium* and *Lachnospiraceae*, thereby maintaining skin microbiome balance and facilitating skin repair. Overall, TAE exhibits functionality in resisting UVB stimulation and protecting the skin. This study innovatively focuses on the aqueous extract of *T. aurantialba*, uncovering its efficacy in combating photoaging. This work not only addresses a significant gap in the research on *T. aurantialba* regarding photoaging, but also establishes a theoretical foundation for the application of TAE in the food and cosmetics industries, while offering crucial references for future research and development efforts. However, the results from metabolomics, transcriptomics, and microbiome analyses have not yet been comprehensively validated empirically. Future work could focus on verifying the accuracy of the relevant pathways (such as lipid metabolism and linoleic acid metabolism) in both *in vitro* and *in vivo* models, and investigating their antibacterial activity against specific microbial communities (such as *Exiguobacterium* and *Lachnospiraceae*). This would further contribute to a more comprehensive elucidation of the mechanism underlying TAE’s photoprotective effects.

## Conclusion

In this study, the main components identified in the TAE were mannitol, acetylcholine, choline, alanine, and other compounds. We found that different concentrations of TAE can promote the migration and repair of Hacat cells and inhibit the expression of ROS. In addition, TAE also showed the potential to repair skin photoaging caused by UVA/UVB light. Metabolomics and transcriptomics analyses reveal that TAE alleviates photoaging effects by modulating skin barrier and inflammation-related pathways. Genes regulated by TAE are primarily enriched in lipid metabolism and circadian rhythms, suggesting that it promotes skin barrier repair and combats photoaging induced by UVA/UVB by reshaping cellular physiological rhythms and lipid metabolic balance. Additionally, skin microbiome analysis shows that TAE boosts *Bacillota* and *Bacteroidota* while inhibiting *Exiguobacterium* and *Lachnospiraceae*, thereby maintaining the balance of the skin microbiome and repairing the skin. In summary, the TAE can resist UVB stimulation and protect the skin. This study provides a theoretical basis for the application of TAE in the food and cosmetics industries, and offers insights for further research and development.

## Figures and Tables

**Table 1:** Composition analysis of TAE.

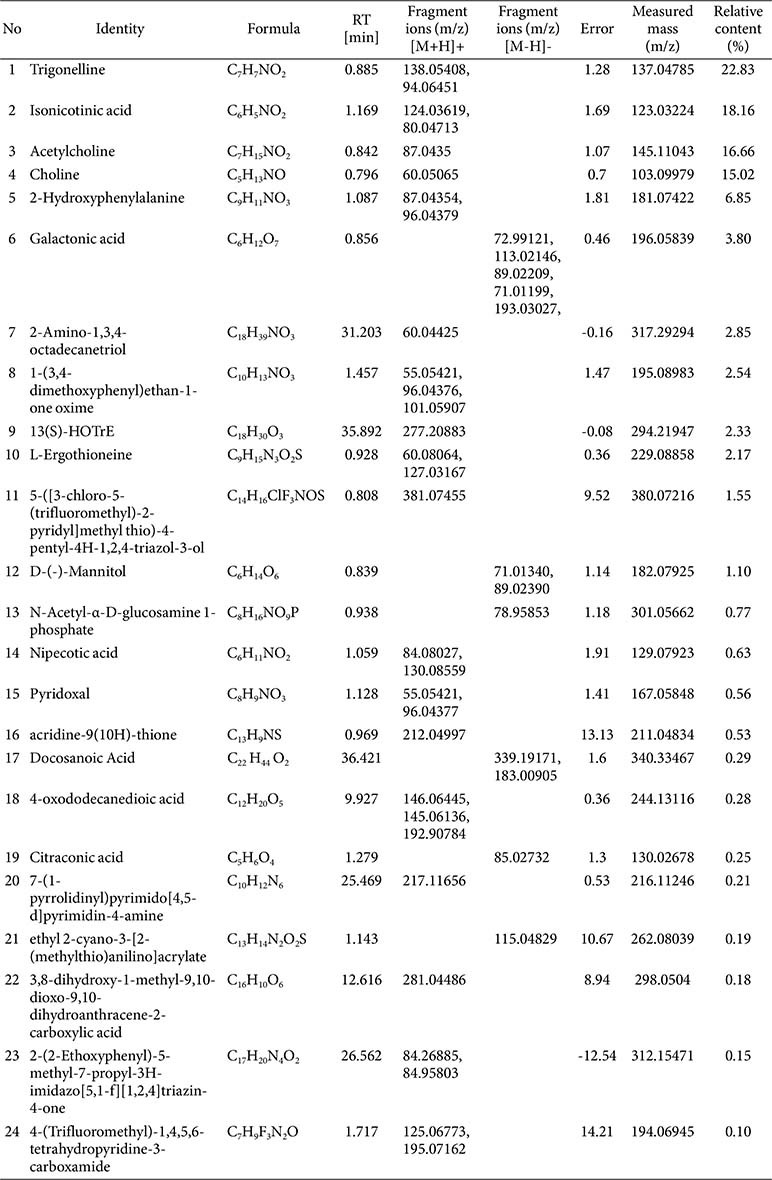

**Table 2:** Expression differences of different bacterial groups.